# Occupational exposures to inorganic dust are associated with emphysema: the SCAPIS cohort

**DOI:** 10.1136/oemed-2025-110140

**Published:** 2025-10-07

**Authors:** Mathias Holm, Linus Schioler, Anna Dahlman-Hoglund, Håkan Tinnerberg, Martin Andersson, Annelie Behndig, Anders Blomberg, Kerstin Cederlund, Jonas Eriksson Ström, Christer Janson, Åse Johnsson, Eva Lindberg, Anders Lindén, Stefan Ljunggren, Andrei Malinovschi, Anna-Carin Olin, Ida Pesonen, Magnus Sköld, Magnus Svartengren, Hanan Tanash, Per Wollmer, Xi-Ming Yuan, Suneela Zaigham, Kjell Torén

**Affiliations:** 1 Department of Occupational and Environmental Medicine, School of Public Health and Community Medicine, Sahlgrenska Academy, University of Gothenburg, Gothenburg, Sweden; 2 Department of Public Health and Clinical Medicine, Umeå University, Umeå, Sweden; 3 Department of Clinical Science, Intervention and Technology, Karolinska Institutet, Stockholm, Sweden; 4 Department of Medical Sciences, Respiratory, Allergy and Sleep Research, Uppsala University, Uppsala, Sweden; 5 Department of Radiology, Sahlgrenska University Hospital, Region Västra Götaland, Gothenburg, Sweden; 6 Department of Radiology, Institute of Clinical Sciences, University of Gothenburg, Gothenburg, Sweden; 7 Division for Lung and Airway Research, Institute of Environmental Medicine, Karolinska Institutet, Stockholm, Sweden; 8 Karolinska Severe COPD Center, Department of Respiratory Medicine and Allergy, Karolinska University Hospital, Stockholm, Sweden; 9 Occupational and Environmental Medicine, Department of Health, Medicine and Caring Sciences, Linköping University, Linköping, Sweden; 10 Department of Medical Sciences, Clinical Physiology, Uppsala University, Uppsala, Sweden; 11 Department of Respiratory Medicine and Allergy, Karolinska University Hospital, Stockholm, Sweden; 12 Division of Immunology and Respiratory Medicine, Department of Medicine, Karolinska Institute, Stockholm, Sweden; 13 Department of Medical Sciences, Occupational and Environmental Medicine, Uppsala University, Uppsala, Sweden; 14 Department of Respiratory Medicine, Skånes universitetssjukhus Malmö, Lund University, Malmö, Sweden; 15 Department of Translational Medicine, Lund University, Malmö, Sweden

**Keywords:** Epidemiology, Dust, Occupational Health

## Abstract

**Objectives:**

There is a lack of knowledge about whether occupational exposures increase the risk of emphysema, especially in never-smokers. Our objective was to determine if occupational exposures are associated with emphysema and impaired diffusing capacity.

**Methods:**

In the Swedish CArdioPulmonary bioImage Study (SCAPIS), persons from the general population aged 50–64 answered a questionnaire and underwent CT of the lung as well as assessment of the diffusing capacity of their lungs for carbon monoxide (DL_CO_), presented as DL_CO_<lower limit of normal (LLN). Emphysema was defined as emphysema in any part of the lungs. Occupational exposures were assessed by a job exposure matrix based on longest held job. ORs with 95% CIs were calculated using logistic multivariable models.

**Results:**

In this cross-sectional study (27 370 persons including 13 981 never-smokers), occupational exposure to inorganic dust was associated with emphysema (OR 1.25, 95% CI 1.07 to 1.47), also among never-smokers, (OR 1.46, 95% CI 1.00 to 2.11). There were associations with DL_CO_<LLN for occupational exposure to inorganic dust and vapour and gases. With all exposures in the same model, inorganic dust was associated with emphysema (OR 1.30, 95% CI 1.08 to 1.57), and vapour and gases were associated with DL_CO_<LLN (OR 1.17, 95% CI 1.00 to 1.38). In those with emphysema and impaired DL_CO_, there was an association with inorganic dust (OR 1.65, 95% CI 1.20 to 2.28), also among never-smokers (OR 3.79, 95% CI 1.35 to 10.63).

**Conclusions:**

Occupational exposures to inorganic dust are associated with emphysema. The association is stronger in those with the combination of emphysema and impaired DL_CO_ indicating serious exposure effects in the alveoli.

**WHAT IS ALREADY KNOWN ON THIS TOPIC**

- There is a lack of knowledge about whether specific occupational exposures increase the risk of emphysema, especially in never-smokers.

**WHAT THIS STUDY ADDS**

- This general population-based study suggests that occupational exposure to inorganic dust is associated with emphysema, also in never-smokers.

**HOW THIS STUDY MIGHT AFFECT RESEARCH, PRACTICE OR POLICY**

- The findings underline the need for further research and preventive actions to reduce this common occupational exposure.

## Introduction

 In high-income countries, the main cause of chronic obstructive pulmonary disease (COPD) is tobacco smoking, but there are several studies showing an association between occupational exposures to dust, fumes and smoke, and COPD.^1 2^ One important comorbidity in COPD is emphysema, which is defined anatomically as destruction of the parenchyma and loss of alveoli. This is different from the operational definitions of COPD which are based on spirometric chronic airflow limitation and presence of certain symptoms. Therefore, COPD and emphysema are distinct and separate disorders. Emphysema can exist without airflow limitation and, likewise, 50% of subjects with COPD do not have emphysema.^3^ In early studies of the disease, emphysema was assessed using either X-rays combined with determination of diffusing capacity or autopsies. In recent years, modern imaging techniques have made it possible to detect early signs of emphysema using CT of the lungs. This technique has been used in a few studies to examine the relation between occupational exposures and emphysema.^4 5^ The ability of the lungs to transfer gas between inhaled air and blood can be assessed by measuring the diffusing capacity of the lungs for carbon monoxide (DL_CO_). It has been proposed that impaired DL_CO_ can reveal mild emphysema not detected by CT.^6^

Tobacco smoking is the number one risk factor for emphysema. However, studies on occupational exposures and risk of emphysema in general populations are scarce, especially in never-smokers. Self-reported occupational exposures to fume and dust were associated with CT-based emphysema in the COPDGene study, which included smokers and former smokers, but not never-smokers.^5^ In the Swedish CArdioPulmonary bioImage Study (SCAPIS) Pilot, an association between self-reported occupational exposure to vapour, gas, dust and fumes (VGDF) and CT-based emphysema was shown, but the sample size was insufficient to separately analyse never-smokers.^7^ Exposure to cadmium has been linked to the development of emphysema, and also to decreased DL_CO_.^8^,^10^ A systematic review on the health problems of dust-exposed coal miners and gold miners concluded that more than 20 years’ work at high levels of dust (compared with miners with less dusty occupations) was associated with more than three times higher odds of having emphysema at autopsy.^11^ No association, however, was found among non-smokers, which was further confirmed in a subsequent study in never-smokers.^12^ Several studies have used self-reported occupational exposure, a measure that is susceptible to recall bias.^13^ The presence of the disease may affect the reporting of the exposure, often causing an overestimation of the exposure. More objective methods, such as job exposure matrices (JEMs), can eliminate or diminish this risk of bias.^14^

The present study aimed to determine whether occupational exposures to inorganic dust, fumes, vapour and gases, and organic dust, assessed using a JEM, are associated with emphysema and impaired DL_CO_, especially in never-smokers.

## Methods

### Study population

Our data were from the SCAPIS, a general population-based study of 30 154 adults aged 50–64 years, randomly selected from the general population.^15 16^ The SCAPIS population was invited to participate at one of six Swedish universities, and data were collected between 2013 and 2018. For the present study, 27 370 participants, 13 981 of whom were never-smokers, with complete information about the longest held occupation, smoking habits and completed pulmonary CT were included. Of these 27 370 participants, 26 439 also had complete information about DL_CO_.

### Clinical investigations

All persons answered a questionnaire, including questions about smoking habits, the longest held occupation and educational level, with completed university degree being the highest of three possible levels. The participants were categorised as current, former or never-smokers. Never-smokers were defined as participants who had smoked fewer than 100 cigarettes in their lifetime.

Dynamic spirometry 15 min after inhalation of 400 µg of salbutamol included forced vital capacity (FVC) and forced expiratory volume at 1 s (FEV_1_), as well as DL_CO_, with the person in a sitting position and using a nose clip. Weight and height were measured. A Jaeger Master Screen PFT system (Vyaire, Mettawa, Illinois, USA) was used for all measurements, which were performed according to American Thoracic Society (ATS)/European Respiratory Society (ERS) standards.^17^ Predicted values or lower limits of normal (LLN) of FEV_1_, FVC and DL_CO_ were based on reference equations from the SCAPIS.^16 18^

The presence of emphysema was based on visual assessment of CT images according to the Fleischner Society Glossary.^15 19^ The extent of emphysema was visually estimated on a 4-point scale (absent, mild, moderate or severe) in the upper, mid and lower zones of each lung.^20^ In the analyses, all subjects with at least mild emphysema in any zone were categorised as having emphysema.^19^

### Occupational exposures

The longest held jobs were coded according to the International Standard Classification of Occupations (ISCO)-88. Based solely on the coding, the occupational exposures to inorganic dust, fumes (smoke from various combustion processes), vapour and gas (substances in the aerosol or the gas phase), and organic dust were assessed using a Swedish JEM.^21^ All exposures were initially graded into low or high (or no exposure) but due to low numbers in the high exposure categories, low exposure and high exposure were merged into any exposure. The occupational exposures were thus graded as exposed or not exposed. Each occupation could be classified as exposed to more than one of the critical agents. There was also a category where all the above occupational exposures were merged into any exposure to VGDF. For further details regarding the JEM, see Torén *et al* 2020.^21^

### Statistical analysis

Demographic characteristics are presented in percentage for categorical variables. Median and IQR were used for continuous variables. Multivariable logistic regression models were used to calculate ORs with 95% CIs of the outcomes associated with different JEM-defined occupational exposures in the whole study population, in ever-smokers and in never-smokers using models for each exposure. One model included all occupational exposures in the same model. Adjustments were made for smoking status (current, former or never-smoker), pack-years, age, sex, study site and educational level. Firth’s bias correction was applied to all models. Age was included as a second-degree polynomial, that is, age and age^2^ were included in the models. Pack-year, where applicable, was modelled with a cubic restricted spline with knots placed at the 5th, 27.5th, 50th, 72.5th and 95th percentiles of the distribution among ever-smokers.

Statistical significance was defined as a p<0.05. Data management and the analyses were conducted using the SAS statistical package, V.9.4M6 (SAS Institute: Inc.).

## Results

### Demographics

The prevalence of emphysema was 5.6% (n=1539), 6.2% (n=819) among men, 5.1% (n=720) among women and 2.1% (n=290) among never-smokers. In current smokers, the prevalence of emphysema was 18% (n=611) and in former smokers 6.4% (n=638). The overall prevalence of DL_CO_<LLN was 8.6% (n=2275), but the prevalence of emphysema and DL_CO_<LLN was only 2.0% (n=465). The main characteristics of the study participants are shown in table 1. More than half of the study population (50.1%) reported that they had never smoked. The prevalence of VGDF exposure was 23.1% (n=6328), 31.8% (n=4209) among men and 15.0% (n=2119) among women (table 1).

**Table 1:** Characteristics of the study population

	All	Men	Women	Emphysema	DL_CO_<LLN	Emphysema+DL_CO_<LLN
N	27 370	13 249 (48.4%)	14 121 (51.6%)	1539 (5.6%)	2275 (8.6%)	465 (2.0%)
Age, years, (IQR)	57.5 (53.7–61.3)	57.5 (53.7–61.3)	57.4 (53.7–61.2)	59.1 (55.1–62.5)	58.2 (54.0–61.7)	59.9 (55.7–62.7)
Women, n	14 121 (51.6%)	n.a.	n.a.	720 (46.8%)	1254 (55.1%)	230 (49.5%)
High educational level, n	12 506 (45.8%)	5468 (41.4%)	7038 (49.9%)	507 (33.1%)	811 (35.8%)	107 (23.2%)
BMI, kg/m^2^ (IQR)	26.3 (23.9–29.3)	26.9 (24.8–29.5)	25.6 (23.0–29.0)	25.5 (55.1–62.5)	26.2 (23.5–29.4)	25.1 (21.9–27.8)
Smoking history						
Current smokers	3399 (12.4%)	1635 (12.3%)	1764 (12.5%)	611 (39.7%)	804 (35.3%)	295 (63.4%)
Former smokers	9990 (36.5%)	4444 (33.5%)	5546 (39.3%)	638 (41.5%)	765 (33.6%)	143 (30.8%)
Never-smokers	13 981 (51.1%)	7170 (54.1%)	6811 (48.2%)	290 (18.8%)	706 (31.0%)	27 (5.8%)
Pack-years	0 (0–12.0)	0 (0–11.5)	0 (0–12.0)	20.0 (4.5–33.0)	12.6 (0–29.0)	30.0 (19.0–38.0)
Pulmonary function						
FEV_1,_ % pred (IQR)	98.2 (89.5–106.5)	98.1 (89.5–106.3)	98.4 (89.6–106.7)	92.0 (80.8–102.0)	87.8 (77.5–96.7)	81.9 (71.5–92.7)
FVC, % pred (IQR)	99.4 (91.3–107.5)	99.3 (91.0–107.3)	99.5 (91.5–107.8)	99.7 (90.6–108.7)	91.5 (83.1–100.8)	95.9 (86.6–106.1)
FEV_1_/FVC<0.70	2572 (9.5%)	1455 (11.1%)	1117 (8.0%)	518 (33.9%)	514 (22.6%)	266 (57.2%)
DL_CO,_ % pred (IQR)	97.8 (88.8–107.0)	97.9 (88.8–107.1)	97.6 (88.6–107.0)	87.8 (75.8–99.4)	74.4 (69.2–77.4)	70.0 (61.8–75.1)
Occupational exposures						
VGDF, n	6328 (23.1%)	4209 (31.8%)	2119 (15.0%)	502 (32.6%)	671 (29.5%)	168 (36.1%)
Inorganic dust, n	2524 (9.2%)	1765 (13.3%)	759 (5.4%)	228 (14.8%)	278 (12.2%)	86 (18.5%)
Fumes, n	3447 (12.6%)	2054 (15.5%)	1393 (9.9%)	268 (17.4%)	361 (15.9%)	94 (20.2%)
Vapour/gas, n	3670 (13.4%)	2336 (17.6%)	1334 (9.4%)	297 (19.3%)	419 (18.4%)	107 (23.0%)
Organic dust, n	1699 (6.2%)	1119 (8.4%)	580 (4.1%)	116 (7.5%)	143 (6.3%)	29 (6.2%)

Data is presented as median and IQR.

BMI, body mass index; DL_CO_, diffusing capacity of the lungs for carbon monoxide; FEV_1_, forced expiratory volume at 1 s; FVC, forced vital capacity; LLN, lower limit of normal; pred, predicted; VGDF, vapour, gas, dust and fumes.

### Emphysema, DL_CO_ <LLN and emphysema with DL_CO_ <LLN

Occupational exposures to inorganic dust, vapour and gases, and fumes were all associated with emphysema when adjusted for smoking status (never, former and current), age, sex and study site (model 1) (table 2). The highest OR was observed for inorganic dust (OR 1.37, 95% CI 1.18 to 1.61). The ORs decreased, and only occupational exposures to inorganic dust were significantly associated with emphysema when adding adjustments for pack-years (model 2) (OR 1.25, 95% CI 1.07 to 1.47). Adding adjustments for educational level did not substantially change these results (model 3, only 53 participants did not have information about educational level).

**Table 2:** OR of CT-based pulmonary emphysema, DL_CO_<LLN and DL_CO_<LLN combined with emphysema and occupational exposures in logistic regression models

Occupational exposures	OR (95% CI)		
	Model 1*	Model 2†	Model 3‡
Emphysema			
VGDF	1.26 (1.12 to 1.42)	1.10 (0.97 to 1.24)	1.06 (0.93 to 1.20)
Inorganic dust	1.37 (1.18 to 1.61)	1.25 (1.07 to 1.47)	1.21 (1.02 to 1.42)
Vapour/gases	1.19 (1.04 to 1.37)	1.04 (0.90 to 1.20)	1.01 (0.87 to 1.17)
Fumes	1.21 (1.05 to 1.40)	1.08 (0.94 to 1.25)	1.05 (0.91 to 1.23)
Organic dust	1.06 (0.86 to 1.30)	1.03 (0.83 to 1.27)	0.99 (0.80 to 1.23)
DL_CO_<LLN			
VGDF	1.25 (1.13 to 1.38)	1.14 (1.03 to 1.27)	1.07 (0.96 to 1.20)
Inorganic dust	1.27 (1.10 to 1.46)	1.19 (1.03 to 1.38)	1.13 (0.98 to 1.31)
Vapour/gases	1.28 (1.14 to 1.44)	1.19 (1.05 to 1.34)	1.13 (0.99 to 1.28)
Fumes	1.16 (1.02 to 1.31)	1.07 (0.94 to 1.22)	1.01 (0.89 to 1.16)
Organic dust	0.94 (0.78 to 1.13)	0.92 (0.76 to 1.11)	0.89 (0.74 to 1.07)
DL_CO_<LLN with emphysema§			
VGDF	1.41 (1.15 to 1.74)	1.12 (0.90 to 1.39)	1.00 (0.79 to 1.25)
Inorganic dust	1.75 (1.35 to 2.26)	1.54 (1.18 to 2.02)	1.40 (1.06 to 1.85)
Vapour/gases	1.38 (1.09 to 1.74)	1.17 (0.92 to 1.50)	1.08 (0.84 to 1.38)
Fumes	1.41 (1.11 to 1.79)	1.21 (0.94 to 1.55)	1.12 (0.86 to 1.45)
Organic dust	0.84 (0.57 to 1.25)	0.78 (0.52 to 1.18)	0.71 (0.47 to 1.09)

* Adjusted for smoking (never, former and current), age, sex and study site.

† Adjusted for smoking (never, former and current), pack-years, age, sex and study site.

‡ Adjusted for smoking (never, former and current), pack-years, age, sex, study site and educational level.

§ DL_CO_<LLN with emphysema compared with no emphysema or DL_CO_<LLN.

DL_CO_, diffusing capacity of the lungs for carbon monoxide; LLN, lower limit of normal; VGDF, vapour, gas, dust and fumes.

In the model adjusted for smoking status, age, sex and study site (model 1), occupational exposures to inorganic dust, vapour and gases and fumes were all associated with DL_CO_<LLN. When adding adjustments for pack-years (model 2), the ORs decreased, and only exposures to inorganic dust (OR 1.19, 95% CI 1.03 to 1.38) and vapour and gases (OR 1.19, 95% CI 1.05 to 1.34) remained statistically significant (table 2). Further adjustments for educational level (model 3, only 48 participants did not have information about educational level) resulted in decreased ORs and CIs including unity.

Analyses of occupational exposures in relation to emphysema with DL_CO_<LLN showed a similar pattern. In the simple model (model 1), occupational exposures to inorganic dust, to vapour and gases, and to fumes were associated with emphysema with DL_CO_<LLN. In the model also adjusted for pack-years (model 2), only the association with exposure to inorganic dust remained statistically significant (OR 1.54, 95% CI 1.18 to 2.02) (table 2). Adding adjustments for educational level (model 3, only 39 participants did not have information about educational level) did not substantially change the association (OR 1.38, 95% CI 1.05 to 1.82).

When including all occupational exposures in the same model, the OR for emphysema was increased only in those exposed to inorganic dust (OR 1.33, 95% CI 1.11 to 1.61) when adjusted for smoking (never, former or current), age, sex and study site (table 3). With further adjustments, the OR decreased to 1.26, 95% CI 1.05 to 1.53, in the most adjusted model (model 3). The other occupational exposures showed no clear association with emphysema (table 3). Furthermore, when all occupational exposures were included in the same model, there were few associations with DL_CO_<LLN, but for emphysema with DL_CO_<LLN, there was a clear association with occupational exposure to inorganic dust, the ORs ranging from 1.54 to 1.73 (table 3).

**Table 3:** OR of CT-based pulmonary emphysema, DL_CO_<LLN and DL_CO_<LLN combined with emphysema and occupational exposures in logistic regression models

Occupational exposures	OR (95% CI)		
	Model 1*	Model 2†	Model 3‡
Emphysema			
Inorganic dust	1.33 (1.11 to 1.61)	1.30 (1.08 to 1.57)	1.26 (1.05 to 1.53)
Vapour/gases	0.99 (0.83 to 1.19)	0.91 (0.76 to 1.09)	0.90 (0.75 to 1.08)
Fumes	1.13 (0.95 to 1.34)	1.07 (0.90 to 1.27)	1.06 (0.89 to 1.26)
Organic dust	0.96 (0.78 to 1.19)	0.96 (0.78 to 1.20)	0.95 (0.76 to 1.18)
DL_CO_<LLN			
Inorganic dust	1.18 (1.00 to 1.39)	1.15 (0.97 to 1.36)	1.12 (0.94 to 1.32)
Vapour/gases	1.23 (1.05 to 1.44)	1.17 (1.00 to 1.38)	1.13 (0.99 to 1.28)
Fumes	0.99 (0.85 to 1.15)	0.95 (0.81 to 1.10)	0.92 (0.79 to 1.08)
Organic dust	0.86 (0.71 to 1.04)	0.87 (0.71 to 1.05)	0.85 (0.70 to 1.03)
DL_CO_<LLN with emphysema§			
Inorganic dust	1.73 (1.26 to 2.37)	1.65 (1.20 to 2.28)	1.54 (1.11 to 2.13)
Vapour/gases	1.00 (0.73 to 1.37)	0.91 (0.66 to 1.25)	0.88 (0.64 to 1.21)
Fumes	1.21 (0.90 to 1.63)	1.12 (0.83 to 1.50)	1.08 (0.80 to 1.45)
Organic dust	0.70 (0.47 to 1.06)	0.70 (0.46 to 1.06)	0.66 (0.44 to 1.01)

All occupational exposures are included in the same model.

* Adjusted for smoking (never, former and current), age, sex and study site.

† Adjusted for smoking (never, former and current), pack-years, age, sex and study site.

‡ Adjusted for smoking (never, former and current), pack-years, age, sex, study site and educational level.

§ DL_CO_<LLN with emphysema compared with no emphysema or DL_CO_<LLN.

DL_CO_, diffusing capacity of the lungs for carbon monoxide; LLN, lower limit of normal.

In general, there were very few statistically significant associations between occupational exposures and emphysema, DL_CO_<LLN, and emphysema with DL_CO_<LLN, when analysing men and women separately. The estimates were on the same level as for the whole population, but with CIs including unity, see online supplemental table S1.

Frequency of occupations classified as exposed to inorganic dust among those with emphysema is presented in online supplemental table S2.

### Never-smokers

The analyses in never-smokers were affected by lower power compared with the analyses in the entire study population. In the single exposure models, the association between occupational exposure to inorganic dust and emphysema remained (OR 1.46, 95% CI 1.00 to 2.11) in the model adjusted for age, sex and study site (model A). When adding educational level into the model, the OR for exposure to inorganic dust decreased (OR 1.41, 95% CI 0.96 to 2.08) (model B). When all occupational exposures were included in the same model, the ORs remained high, but with CIs including unity (table 4). The other exposures did not show any clear associations.

**Table 4:** OR of CT-based pulmonary emphysema, DL_CO_<LLN and DL_CO_<LLN combined with emphysema and occupational exposures in logistic regression models in never-smokers

Occupational exposures	OR (95% CI)					
	Emphysema		DL_CO_<LLN		DL_CO_<LLN with emphysema*	
	Model A†	Model B‡	Model A†	Model B‡	Model A†	Model B‡
Never-smokers						
Vapour, gas, dust and fumes	1.19 (0.90 to 1.57)	1.18 (0.87 to 1.59)	1.18 (0.98 to 1.43)	1.09 (0.89 to 1.34)	1.90 (0.89 to 4.05)	1.65 (0.72 to 3.80)
Inorganic dust	1.46 (1.00 to 2.11)	1.41 (0.96 to 2.08)	1.32 (1.02 to 1.73)	1.24 (0.94 to 1.62)	3.40 (1.46 to 7.90)	2.79 (1.13 to 6.89)
Vapour/gases	0.97 (0.67 to 1.41)	0.95 (0.65 to 1.40)	1.13 (0.89 to 1.44)	1.05 (0.82 to 1.34)	0.72 (0.21 to 2.46)	0.67 (0.20 to 2.23)
Fumes	1.00 (0.69 to 1.45)	1.00 (0.68 to 1.45)	1.10 (0.87 to 1.40)	1.02 (0.79 to 1.30)	2.30 (0.99 to 5.30)	2.38 (1.01 to 5.62)
Organic dust	1.47 (0.96 to 2.25)	1.41 (0.96 to 2.08)	1.16 (0.85 to 1.59)	1.08 (0.78 to 1.49)	3.04 (1.17 to 7.93)	2.25 (0.78 to 6.45)
Never-smokers (all occupational exposures in the same model)						
Inorganic dust	1.53 (0.98 to 2.38)	1.48 (0.94 to 2.31)	1.29 (0.95 to 1.77)	1.26 (0.92 to 1.72)	3.79 (1.35 to 10.63)	3.31 (1.17 to 9.37)
Vapour/gases	0.79 (0.48 to 1.28)	0.80 (0.49 to 1.29)	1.01 (0.74 to 1.38)	0.98 (0.71 to 1.34)	0.16 (0.04 to 0.64)	0.18 (0.05 to 0.69)
Fumes	1.01 (0.64 to 1.58)	1.01 (0.64 to 1.59)	1.02 (0.76 to 1.38)	0.97 (0.72 to 1.31)	3.77 (1.46 to 9.69)	3.92 (1.51 to 10.18)
Organic dust	1.32 (0.84 to 2.09)	1.29 (0.81 to 2.06)	1.06 (0.76 to 1.47)	1.02 (0.73 to 1.42)	2.08 (0.69 to 6.34)	1.74 (0.55 to 5.52)

Exposed never-smokers vs non-exposed never-smokers.

* DL_CO_<LLN with emphysema compared with no emphysema or DL_CO_<LLN.

† Adjusted for age, sex and study site.

‡ Adjusted for age, sex, study site and educational level.

DL_CO_, diffusing capacity of the lungs for carbon monoxide; LLN, lower limit of normal.

Occupational exposure to inorganic dust was associated with DL_CO_<LLN among never-smokers (OR 1.32, 95% CI 1.02 to 1.73). For emphysema with DL _CO_<LLN, there was a clear association with exposure to inorganic dust (OR 3.40, 95% CI 1.46 to 7.90) (model A). When all exposures were included in the same model, occupational exposures to inorganic dust (OR 3.79, 95% CI 1.35 to 10.63) and fumes (OR 3.77, 95% CI 1.46 to 9.69) were clearly associated with emphysema with DL_CO_<LLN (model A) (table 4).

### Ever-smokers

In these analyses, we merged current smokers and former smokers into the category of ever-smoker to get enough statistical power. In the single exposure models, emphysema was associated with occupational exposure to inorganic dust (OR 1.20, 95% CI 1.01 to 1.43) after adjustments for age, sex, study site and pack-years (model C). Further adjustments also for educational level (model D) resulted in a decreased and statistically non-significant OR for inorganic dust (OR 1.16, 95% CI 0.96 to 1.39). When all occupational exposures were included in the same model, the OR for inorganic dust was statistically significant (OR 1.24, 95% CI 1.01 to 1.53) (model C) (table 5).

**Table 5:** OR of CT-based pulmonary emphysema, DL_CO_<LLN and DL_CO_<LLN combined with emphysema and occupational exposures in logistic regression models in ever-smokers

Occupational exposures	OR (95% CI)					
	Emphysema		DL_CO_<LLN		DL_CO_<LLN with emphysema*	
	Model C†	Model D‡	Model C†	Model D‡	Model C†	Model D‡
Ever-smokers						
Vapour, gas, dust and fumes	1.07 (0.93 to 1.23)	1.03 (0.89 to 1.19)	1.12 (0.99 to 1.27)	1.06 (0.93 to 1.21)	1.08 (0.86 to 1.35)	0.97 (0.76 to 1.23)
Inorganic dust	1.20 (1.01 to 1.43)	1.16 (0.96 to 1.39)	1.14 (0.96 to 1.35)	1.09 (0.91 to 1.29)	1.47 (1.11 to 1.94)	1.35 (1.01 to 1.80)
Vapour/gases	1.05 (0.90 to 1.23)	1.01 (0.86 to 1.19)	1.21 (1.05 to 1.39)	1.15 (1.00 to 1.34)	1.21 (0.94 to 1.55)	1.10 (0.85 to 1.43)
Fumes	1.10 (0.93 to 1.29)	1.07 (0.90 to 1.25)	1.06 (0.91 to 1.23)	1.02 (0.87 to 1.19)	1.16 (0.89 to 1.51)	1.07 (0.82 to 1.40)
Organic dust	0.94 (0.74 to 1.19)	0.91 (0.72 to 1.16)	0.82 (0.66 to 1.04)	0.81 (0.64 to 1.02)	0.68 (0.44 to 1.06)	0.65 (0.42 to 1.02)
Ever-smokers (all occupational exposures in the same model)						
Inorganic dust	1.24 (1.01 to 1.53)	1.21 (0.98 to 1.49)	1.09 (0.89 to 1.32)	1.06 (0.87 to 1.29)	1.53 (1.10 to 2.14)	1.45 (1.03 to 2.03)
Vapour/gases	0.93 (0.76 to 1.14)	0.91 (0.75 to 1.12)	1.23 (1.03 to 1.48)	1.20 (0.99 to 1.44)	1.00 (0.73 to 1.39)	0.96 (0.69 to 1.33)
Fumes	1.08 (0.90 to 1.31)	1.07 (0.88 to 1.29)	0.93 (0.78 to 1.11)	0.91 (0.76 to 1.09)	1.04 (0.76 to 1.41)	1.00 (0.73 to 1.36)
Organic dust	0.89 (0.70 to 1.14)	0.88 (0.69 to 1.12)	0.79 (0.62 to 1.00)	0.78 (0.62 to 0.99)	0.62 (0.40 to 0.97)	0.61 (0.39 to 0.96)

Exposed ever-smokers versus non-exposed ever-smokers.

* DL_CO_<LLN with emphysema compared with no emphysema or DL_CO_<LLN.

† Adjusted for pack-years, age, sex and study site.

‡ Adjusted for pack-years, age, sex, study site and educational level.

DL_CO_, diffusing capacity of the lungs for carbon monoxide; LLN, lower limit of normal.

In the model adjusted for age, sex, study site and pack-years (model C), occupational exposure to vapour and gases was related to DL_CO_<LLN (OR 1.21, 95% CI 1.05 to 1.39). For emphysema with DL_CO_<LLN, there was a distinct association with exposure to inorganic dust (OR 1.47, 95% CI 1.11 to 1.94) (table 5). When all occupational exposures were included in the same model, occupational exposure to vapour and gases was still related to DL_CO_<LLN (OR 1.23, 95% CI 1.03 to 1.48), and occupational exposure to inorganic dust showed a clear association with emphysema with DL_CO_<LLN (OR 1.53, 95% CI 1.10 to 2.14) (model C) (table 5).

## Discussion

This study aimed to investigate the association between occupational exposures and emphysema, alone or in combination with impaired diffusing capacity. The main finding was a clear association between occupational exposure to inorganic dust and emphysema, with or without impaired diffusing capacity. The association was present in both never-smokers and ever-smokers.

We analysed four different occupational exposure modalities. Exposure to inorganic dust was the modality that most consistently was associated with emphysema in the whole sample (ORs ranging from 1.21 to 1.37) and in ever-smokers (ORs ranging from 1.16 to 1.24). Among never-smokers, the ORs ranged from 1.41 to 1.53, but with CIs including unity. Hence, we consider occupational exposure to inorganic dust to be associated with increased odds for emphysema.

Our observations of an association between occupational exposure to inorganic dust and emphysema are supported by earlier literature. Exposure to inorganic dust in the form of cadmium dust, coal dust or silica dust has been linked to emphysema in small occupational cohort studies in men, but also in large cohort studies of gold and coal miners.^8 11 22 23^ In a more recent study in current and former smokers, using quantitative measurement of emphysema on CT imaging, self-reported occupational exposures to dust and fumes combined were associated with emphysema.^5^ Self-reported exposure to VGDF was also associated with emphysema on CT imaging in the general population-based Swedish SCAPIS Pilot, but there was a lack of statistical power to separately analyse never-smokers.^7^

We also analysed the association between occupational exposures and emphysema with DL_CO_<LLN, a condition that comprises emphysema with impaired gas exchange, indicating that the alveoli are seriously affected. We consider this entity to be a more severe variety of emphysema. In this group, we observed increased ORs in relation to exposure to inorganic dust, most clearly in never-smokers but also in ever-smokers. In never-smokers, we also found a clear association with occupational exposure to fumes. The exposure modality ‘fumes’ in this JEM classification is smoke from various combustion processes, which means an aerosol of very fine particles, that is, an exposure that could reach the very distal airways and alveoli. It is an exposure quite similar to that of tobacco smoke. Hence, we consider it quite plausible that exposure to fumes can be associated with emphysema with impaired diffusing capacity.

It has been previously suggested that men and women may react differently when exposed to dust and smoke.^24^ It has also been suggested that women could be more sensitive to irritating exposures. In our study, the ORs for emphysema, also combined with impaired diffusing capacity, were similar in men and women. The results, therefore, do not support the opinion that women are more vulnerable than men to the exposures in question. However, one possible explanation for the results could be that the same occupational title implies different exposure levels in men and women or that women and men in general have different occupations classified as exposed, for example, to inorganic dust.^25 26^ It should also be noted that women, compared with men, were less exposed to VGDF. Nevertheless, women are also at risk of developing occupational lung diseases. It underlines the importance of obtaining a detailed occupational history in both women and men.^5^

Emphysema is very strongly associated with exposure to tobacco smoke. Even with rigorous controlling for smoking, residual confounding due to smoking may persist. One option to avoid this problem is to analyse never-smokers, and an obvious strength of our study is the high number of never-smokers. Despite our large study population, however, the study seems to be somewhat under-powered since the outcomes were relatively rare in the never-smokers. The prevalence of emphysema was 2.1% among never-smokers, compared with 9.3% among ever-smokers. Emphysema combined with DL_CO_<LLN was seen in only 27 never-smokers. As already mentioned, for never-smokers, there were increased ORs for emphysema in relation to occupational exposures to inorganic dust, but the CIs included unity. However, the entity of emphysema with DL_CO_<LLN was clearly associated with occupational exposure to inorganic dust in never-smokers, which supports the association between inorganic dust and emphysema.

Based on our recently developed JEM, occupational exposure to any VGDF was 23% in the entire study population and 20% in never-smokers. Nine per cent were exposed to inorganic dust, the occupational exposure modality that was most consistently associated with emphysema. These figures are smaller compared with data from similar JEM-based general population studies in the European Community Respiratory Health Survey cohort, the Swiss Study on Air Pollution and Lung Diseases in Adults cohort and the UK Biobank cohort where figures up to twice as high have been presented.^27^,^30^ This could partly be explained by the fact that our study assessed the longest held job and not a full job history. It has also been suggested that high socioeconomic status is associated with participation in the SCAPIS, which implies a lower rate of occupational exposure to VGDF among the participants as this exposure is closely connected to low socioeconomic status.^31^ Typical occupations associated with high exposure to inorganic dust include mining, quarrying, blasting, concrete casting and insulation.^21^

In the present study, we have adjusted for educational level to control for socioeconomic status, but adjustment for socioeconomic status in occupational epidemiology is a controversial issue. If socioeconomic status is a confounder, that is, a predictor for both exposure and outcome, there is a reason for adjustment. On the other hand, if certain occupational exposures such as inorganic dust or VGDF are closely related to educational level, this might cause overadjustments and potentially affect the validity of the obtained estimates. Hence, we present models with and without educational level.

The present study is unique in the sense that we performed assessment of specific occupational exposures using a JEM. The use of a JEM is a way of assessing occupational exposures in a transparent and systematic way by transforming job titles into specific occupational exposures. The use of a JEM minimises the risk of information bias compared with individually self-reported exposures.^13 32^ On the other hand, exposures derived from JEMs can create an exposure misclassification, and as both diseased and non-diseased participants will be assigned the same exposure for a given occupation, this will result in a non-differential misclassification. This introduces a Berkson type error that will not bias the risk estimates but only lead to reduced power of the study in linear regression models.^14^ In studies like ours, with both exposure and outcome on a dichotomous scale, it is not evident that the risk estimates are unbiased.

The most obvious limitation of our study is the cross-sectional nature of the data. This rules out the possibility to make time-dependent analyses and claim causal inference due to the inability to judge which came first, the exposure or the outcome. Another limitation is that the occupational exposure was based on the longest held job and not a complete job history, which means that participants classified as not exposed may have had another job that would have classified them as exposed. This could have introduced non-differential misclassification and attenuation of observed associations. Future studies should include full occupational histories to enable a comprehensive assessment of occupational exposure. Finally, the age span is narrow, 50–64 years, which hampers generalisation of the results to other age groups. Strengths of our study include a large sample from the general population with a high proportion of never-smokers, objective JEM-based assessments of specific occupational exposures and a high-quality definition of emphysema based on visual assessment of CT images.

In conclusion, occupational exposures, most importantly those to inorganic dust, are associated with emphysema in both ever-smokers and never-smokers. The associations are stronger in those with a combination of emphysema and impaired DL_CO_, indicating serious exposure effects in the alveolar region.

## Supplementary material

10.1136/oemed-2025-110140 online supplemental table 1

10.1136/oemed-2025-110140 online supplemental table 2

## Data Availability

Data are available on reasonable request.
