# Supplementary material for: Occupational exposures to inorganic dust are associated with emphysema: the SCAPIS cohort
Source: Occup Environ Med. 2025 Oct 7;82(9):e110140. doi: 10.1136/oemed-2025-110140 (PMC12703276; doi:10.1136/oemed-2025-110140)
Supplement: online supplemental table 1 [file oemed-82-9-s001.docx]

**Table S1. Odds ratio (OR) of computed tomography**–**based pulmonary emphysema, DL_CO_ <LLN and DL_CO_ <LLN combined with emphysema and occupational exposures in logistic regression models.**

| **Occupational exposures** |  |  |  |
| --- | --- | --- | --- |
|  | **Model 1^*^** | **Model 2**^†^ | **Model 3^‡^** |
| **OR (95% confidence interval)** | | | |
| **Men Emphysema** | | | |
| Vapor, gas, dust, and fumes (VGDF) | 1.28 (1.10–1.48) | 1.14 (0.97–1.33) | 1.09 (0.92–1.29) |
| Inorganic dust | 1.35 (1.12–1.63) | 1.26 (1.04–1.53) | 1.21 (0.99–1.48) |
| Vapor/gases | 1.13 (0.95–1.35) | 1.01 (0.85–1.21) | 0.97 (0.81–1.17) |
| Fumes | 1.17 (0.97–1.41) | 1.09 (0.90–1.32) | 1.07 (0.88–1.30) |
| Organic dust | 1.02 (0.80–1.31) | 1.02 (0.80–1.31) | 0.97 (0.75–1.25) |
| **Women Emphysema** | | | |
| VGDF | 1.24 (1.02–1.50) | 1.03 (0.85–1.26) | 1.01 (0.82–1.24) |
| Inorganic dust | 1.46 (1.11–1.93) | 1.25 (0.94–1.67) | 1.21 (0.90–1.63) |
| Vapor/gases | 1.28 (1.03–1.61) | 1.10 (0.87–1.38) | 1.07 (0.84–1.36) |
| Fumes | 1.27 (1.02–1.58) | 1.04 (0.83–1.31) | 1.01 (0.80–1.28) |
| Organic dust | 1.15 (0.80–1.67) | 1.04 (0.71–1.53) | 1.04 (0.71–1.53) |
| **Men DL_CO_ <LLN** | | | |
| VGDF | 1.18 (1.03–1.35) | 1.08 (0.94–1.25) | 1.07 (0.92–1.25) |
| Inorganic dust | 1.22 (1.02–1.46) | 1.17 (0.97–1.40) | 1.15 (0.95–1.38) |
| Vapor/gases | 1.31 (1.12–1.54) | 1.23 (1.05–1.45) | 1.23 (1.04–1.45) |
| Fumes | 1.08 (0.91–1.28) | 1.02 (0.85–1.21) | 1.00 (0.84–1.20) |
| Organic dust | 0.83 (0.65–1.05) | 0.82 (0.65–1.05) | 0.80 (0.63–1.03) |
| **Women DL_CO_ <LLN** | | | |
| VGDF | 1.33 (1.14–1.55) | 1.21 (1.04–1.42) | 1.11 (0.95–1.31) |
| Inorganic dust | 1.34 (1.06–1.69) | 1.24 (0.98–1.57) | 1.15 (0.90–1.47) |
| Vapor/gases | 1.23 (1.02–1.48) | 1.13 (0.94–1.36) | 1.02 (0.84–1.24) |
| Fumes | 1.26 (1.05–1.51) | 1.13 (0.94–1.36) | 1.01 (0.80–1.28) |
| Organic dust | 1.13 (0.85–1.51) | 1.09 (0.81–1.46) | 1.07 (0.79–1.43) |
| **Men DL_CO_ <LLN with emphysema^#^** | | | |
| VGDF | 1.30 (0.99–1.72) | 1.05 (0.79–1.40) | 0.94 (0.69–1.28) |
| Inorganic dust | 1.64 (1.18–2.27) | 1.54 (1.09–2.16) | 1.42 (1.00–2.01) |
| Vapor/gases | 1.28 (0.94–1.74) | 1.15 (0.83–1.58) | 1.08 (0.78–1.50) |
| Fumes | 1.39 (1.00–1.93) | 1.27 (0.90–1.78) | 1.20 (0.85–1.70) |
| Organic dust | 0.59 (0.35–1.00) | 0.55 (0.32–0.95) | 0.49 (0.28–0.86) |
| **Women DL_CO_ <LLN with emphysema^#^** | | | |
| VGDF | 1.59 (1.16–2.17) | 1.21 (0.88–1.69) | 1.07 (0.76–1.50) |
| Inorganic dust | 2.06 (1.36–3.12) | 1.61 (1.03–2.51) | 1.40 (0.88–2.23) |
| Vapor/gases | 1.55 (1.09–2.21) | 1.20 (0.83–1.75) | 1.06 (0.72–1.56) |
| Fumes | 1.44 (1.01–2.06) | 1.10 (0.76–1.61) | 0.94 (0.64–1.39) |
| Organic dust | 1.61 (0.90–2.88) | 1.44 (0.78–2.64) | 1.46 (0.79–2.68) |

*Adjusted for smoking (never, former, and current), age, and study site.

† Adjusted for smoking (never, former, and current), pack-years, age, and study site.

**^‡^** Adjusted for smoking (never, former, and current), pack-years, age, study site, and educational level.

^#^DL_CO_ <LLN with emphysema compared with no emphysema or DL_CO_ <LLN.

DL_CO_ = diffusing capacity of the lungs for carbon monoxide; LLN = lower limit of normal
