# Supplementary material for: Occupational exposures to inorganic dust are associated with emphysema: the SCAPIS cohort
Source: Occup Environ Med. 2025 Oct 7;82(9):e110140. doi: 10.1136/oemed-2025-110140 (PMC12703276; doi:10.1136/oemed-2025-110140)
Supplement: online supplemental table 2 [file oemed-82-9-s002.docx]

**Table S2. Frequency of occupations classified as exposed to inorganic dust among those with computer tomography-based pulmonary emphysema.**

| **ISCO STANDARD DESCRIPTION** | | | | |
| --- | --- | --- | --- | --- |
| **ISCO_STANDARD_DESCRIPTION** | **Frequency** | **Percent** | **Cumulative Frequency** | **Cumulative Percent** |
| **Archivists and curators** | 1 | 0.44 | 1 | 0.44 |
| **Armed forces** | 3 | 1.32 | 4 | 1.75 |
| **Blacksmiths, hammer-smiths and forging-press workers** | 2 | 0.88 | 6 | 2.63 |
| **Bricklayers and stonemasons** | 2 | 0.88 | 8 | 3.51 |
| **Building caretakers** | 11 | 4.82 | 19 | 8.33 |
| **Building frame and related trades workers not elsewhere classified** | 22 | 9.65 | 41 | 17.98 |
| **Concrete placers, concrete finishers and related workers** | 3 | 1.32 | 44 | 19.30 |
| **Dairy and livestock producers** | 2 | 0.88 | 46 | 20.18 |
| **Domestic helpers and cleaners** | 29 | 12.72 | 75 | 32.89 |
| **Earth-moving- and related plant operators** | 2 | 0.88 | 77 | 33.77 |
| **Electrical-equipment assemblers** | 15 | 6.58 | 92 | 40.35 |
| **Glaziers** | 1 | 0.44 | 93 | 40.79 |
| **Heavy truck and lorry drivers** | 16 | 7.02 | 109 | 47.81 |
| **Helpers and cleaners in offices, hotels and other establishments** | 5 | 2.19 | 114 | 50.00 |
| **Librarians and related information professionals** | 10 | 4.39 | 124 | 54.39 |
| **Lifting-truck operators** | 8 | 3.51 | 132 | 57.89 |
| **Machine-tool operators** | 16 | 7.02 | 148 | 64.91 |
| **Messengers, package and luggage porters and deliverers** | 15 | 6.58 | 163 | 71.49 |
| **Metal finishing-, plating- and coating-machine operators** | 1 | 0.44 | 164 | 71.93 |
| **Miners and quarry workers** | 1 | 0.44 | 165 | 72.37 |
| **Ore and metal furnace operators** | 1 | 0.44 | 166 | 72.81 |
| **Painters and related workers** | 13 | 5.70 | 179 | 78.51 |
| **Plumbers and pipe fitters** | 7 | 3.07 | 186 | 81.58 |
| **Precision-instrument makers and repairers** | 2 | 0.88 | 188 | 82.46 |
| **Sculptors, painters and related artists** | 5 | 2.19 | 193 | 84.65 |
| **Sheet metal workers** | 8 | 3.51 | 201 | 88.16 |
| **Telegraph and telephone installers and servicers** | 2 | 0.88 | 203 | 89.04 |
| **Tree and shrub crop growers** | 7 | 3.07 | 210 | 92.11 |
| **Welders and flamecutters** | 18 | 7.89 | 228 | 100.00 |
